# Ectoparasitic fungi *Rickia wasmannii* infection is associated with smaller body size in *Myrmica* ants

**DOI:** 10.1038/s41598-021-93583-0

**Published:** 2021-07-13

**Authors:** Sándor Csősz, Zoltán Rádai, András Tartally, Lilla Erika Ballai, Ferenc Báthori

**Affiliations:** 1 Evolutionary Ecology Research Group, Institute of Ecology and Botany, Centre for Ecological Research, Alkotmány út 2-4, Vácrátót, 2163 Hungary; 2 MTA-ELTE-MTM Ecology Research Group, Pázmány Péter sétány 1/C, Budapest, 1117 Hungary; 3 Lendület Seed Ecology Research Group, Institute of Ecology and Botany, Centre for Ecological Research, Alkotmány út 2-4, Vácrátót, 2163 Hungary; 4 Department of Evolutionary Zoology and Human Biology, University of Debrecen, Egyetem tér 1, Debrecen, 4032 Hungary

**Keywords:** Biodiversity, Coevolution, Social evolution

## Abstract

Parasitism-generated negative effects on ant societies are multifaceted, implying individual and colony-level responses. Though laboratory based evidence shows that the sublethal fungus *Rickia wasmannii* is responsible for physiological and behavioral responses that may negatively affect individual workers’ resilience and life expectancy in *Myrmica* ant workers, colony-level stress response to this parasite is largely unknown. Here, we focus on understanding of a long-term, colony-level effect of *Rickia* infection on *Myrmica scabrinodis* ant populations by tracking trait size-based changes. We collected worker specimens from infected and uninfected colonies from the same population in order to: (1) compare body size in response to parasitism, (2) assess the extent to which possible changes in size are associated with the severity of infection, and (3) investigate shifts in body size in response to infection over time by testing correlation of workers’ ages and sizes. We found that workers from infected colonies were significantly smaller than their healthy congeners, but neither infection level nor the age of the workers showed significant correlation with the size in infected colonies. Decreasing body sizes in infected colonies can be ascribed to workers’ mediated effect toward developing larvae, which are unable to attain the average body size before they pupate.

## Introduction

Ants (Hymenoptera: Formicidae), the most widespread social organisms on Earth, attract an amazing diversity of parasitic organisms, such as viruses^1^, bacteria^2^, fungi^3,4^, and an array of uni- and multicellular animal organisms^5,6^. Many of these parasites cause lethal diseases^7^, but most are sublethal, i.e. they do not necessarily pose an imminent danger, though they are assumed to have detrimental effects on the quality of the hosts’ lives^4,8^.

The ectoparasitic fungus, *Rickia wasmannii* Cavara, 1899 (Ascomycota: Laboulbeniales) is a typical sublethal parasite of several *Myrmica* (Hymenoptera: Formicidae) species which has long been believed to have no detrimental effect on its host individuals^9–11^. For a century, very little was known about this parasitic organism, and only scant information was available on its distribution and host specificity^4,12,13^. The effect of the fungus on the physiology of its host species was largely unknown. In recent years, modern research has shed light on the real nature of this organism and has shown that it has negative effects on individual host ants. Based on previous studies on the interactions between *R. wasmannii* and the main host *Myrmica scabrinodis* Nylander, 1846, infected workers show higher mortality under laboratory conditions^14,15^. Infected *M. scabrinodis* workers were also shown to exhibit improved sanitary behavior^14^, decreased level of intraspecific aggression^16,17^ and reduced cuticle thickness^18^ which may be disadvantageous for the infected colonies in competitive interactions.

Although *R. wasmannii* is known to elicit different detrimental responses in individual workers, colonies seem to resist and compensate for the negative effects of the infection; they contain queens, rear larvae and pupae, and all age-classes of workers are present (see Csata et al.^17^). This may be due to the fact that it is often challenging to study colony-level effects of an infection under in situ conditions, particularly when complex environmental parameters and multifactorial relationships with a number of other organisms^8,15,19^ must be taken into consideration. This may be one of the reasons why a colony-level stress effect of *Rickia* fungal parasites has never been the subject of focused research.

Our current investigation, therefore, differs from naïve approaches and is focusing on indirect evidence. We hypothesized that the colony-level negative effect of the infection is detectable through the decline in size of colony workers. Indeed, resource allocation and colony growth^20,21^ are influenced by an array of environmental factors, e.g. temperature^22^, social structure, starvation^23^ and parasitism^21,24^ in social insects. Indeed, the ability of nestmate workers to regulate development of next generation of larvae could be reduced in response to environmental stressors^25^ such as parasitism mediated stress. This constitutes the basis of our concept. We speculate that the infection has detrimental effects on the brood caring workers, making them unable to engage in foraging activity, feeding and grooming behavior toward their larvae to the necessary extent, and the declining larval growth rate ultimately leads to a decrease in the size of the next generation of workers. *R. wasmannii* infection is transferred over generations, causing a long-term chronic, life-long infection in a colony, which is known to expand year by year in the nest with some level of seasonal fluctuation^26^. The detrimental colony-level effect of the infection accumulated over time can be measured in workers’ sizes. We hypothesized that if *R. wasmannii* infection is widespread in an ant colony, it will detrimentally affect the size of workers groomed by infected sisters owing to the parental colony’s reduced fitness. To test this hypothesis, we compared the sizes of randomly sampled workers from infected and uninfected colonies.

How can we rule out the possibility that the size decline is caused not by direct larval infection but by mediated colony-level stress among workers? *Rickia wasmannii* fungus is found to grow on imago^14,27^ and is not known to infect ant larvae^11,28^ but in order to make sure that a possible size decline is ascribed to stress mediated by nursing workers and is not caused by direct larval infections, trait scaling patterns were also observed. The background of this approach is that infections in the larval stage often cause scrambled trait combinations in adults^29^ via altered static trait allometries. If significant shifts in trait scaling were detected in infected colonies, parasitism generated stress might not only be mediated by infected workers.

We also tested whether the extent of infection (i.e., number of thalli on the cuticular surface of the ants) and the workers’ ages affect the detected changes in imaginal size. Therefore, we registered both infection level and estimated age of the workers in the colony.

## Results

**Figure 1:**
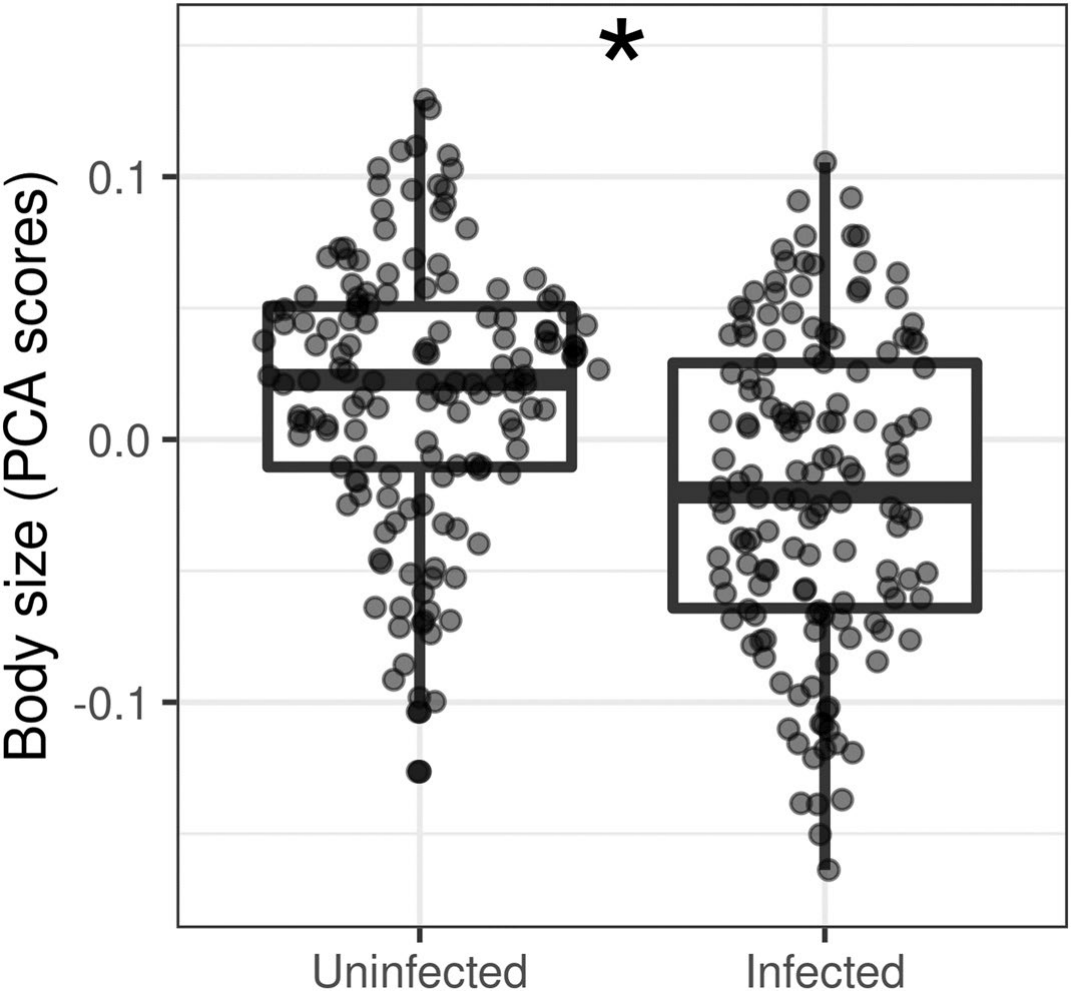
Distribution of PCA axis values representing body size in uninfected and infected colonies. Asterisk marks statistically significant difference.

**Figure 2:**
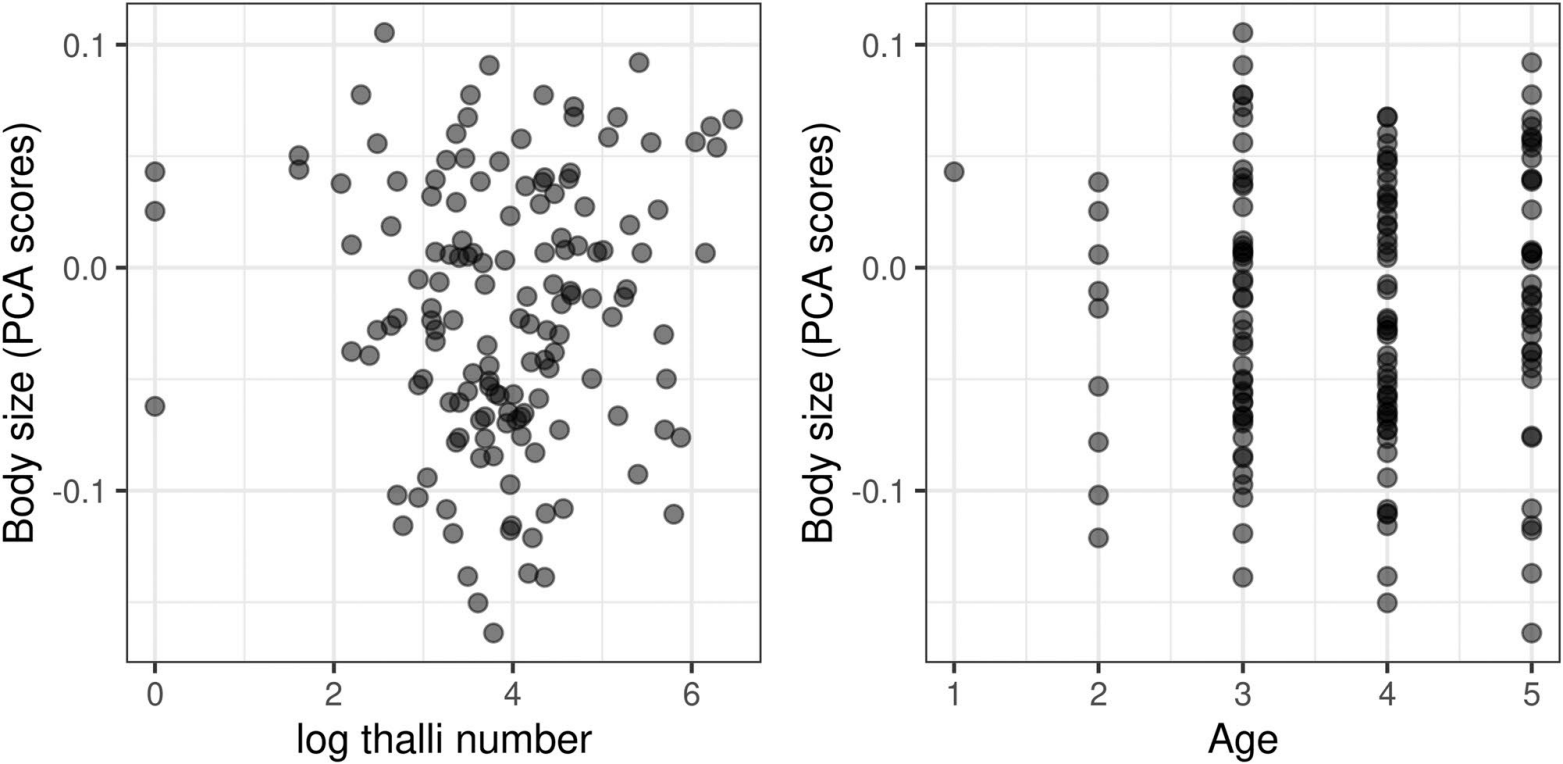
Visualization of how log-transformed thalli number (left panel) and age (right panel) are associated with body size; based on our LMM neither of them had significant effect on the workers' size.

**Figure 3:**
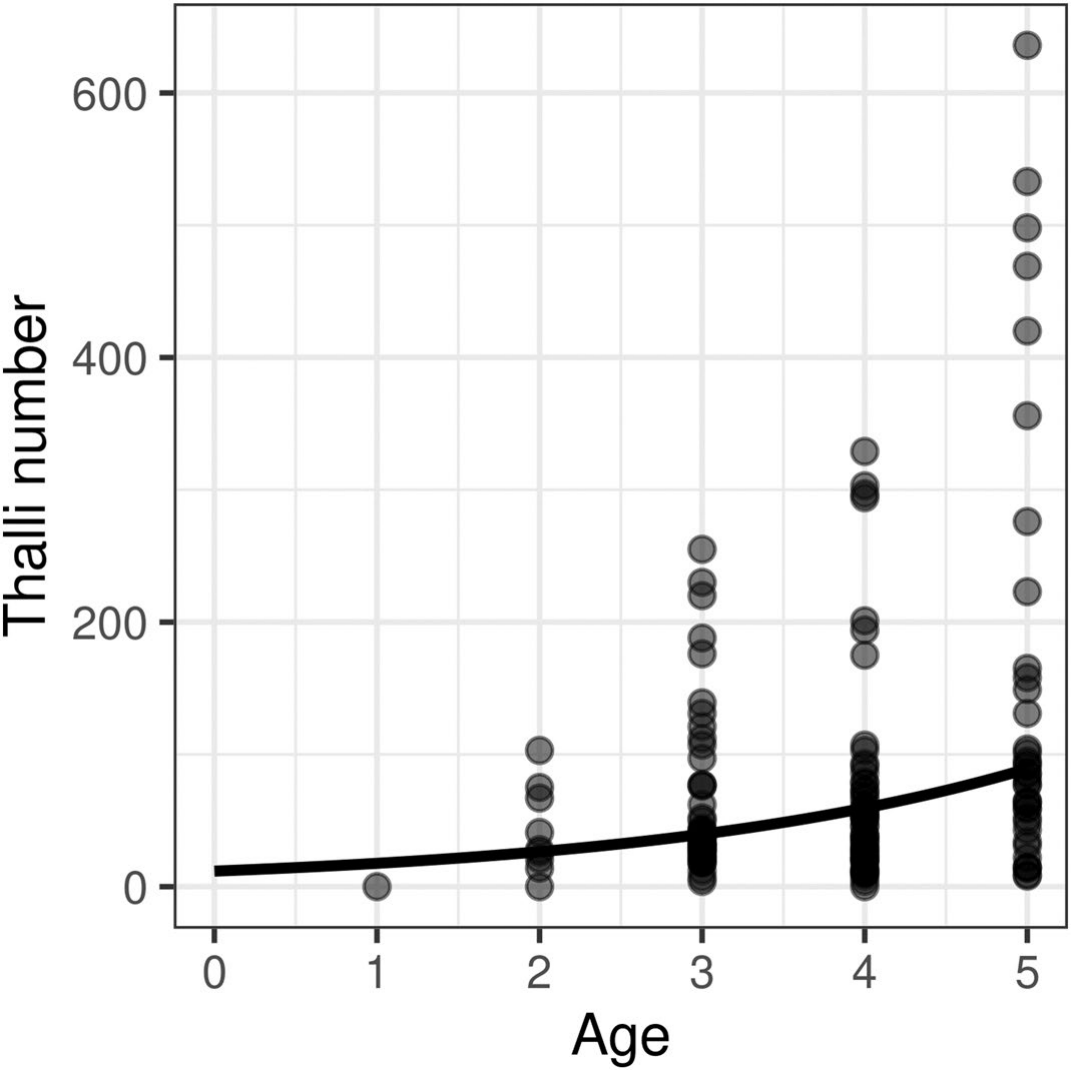
Association of age and thalli number of workers among infected colonies. The solid line represents the association predicted on the Poisson GLMM.

**Figure 4:**
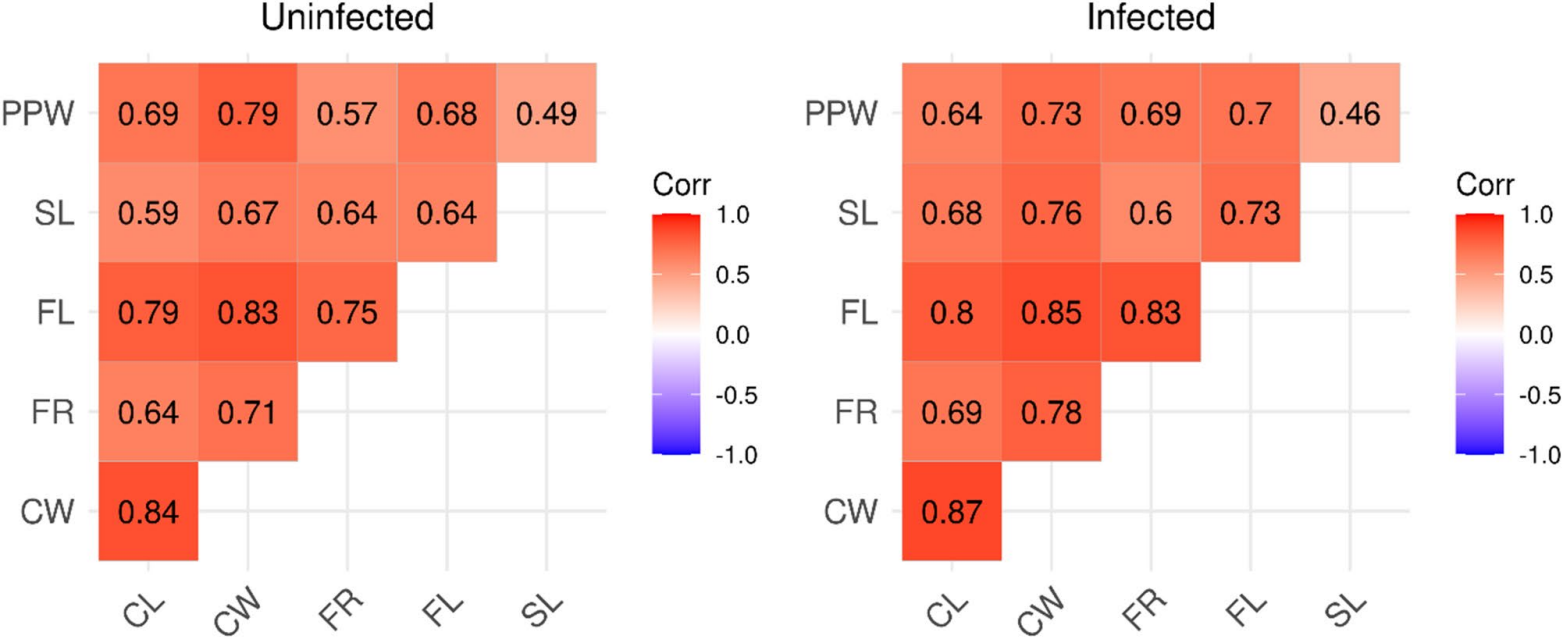
Correlograms visualizing the trait associations of measured body size variables in uninfected (left panel) and infected (right panel) colonies.

A total of 300 workers from 30 colonies were measured (15 colonies of each class). The calculated Intra Class Correlation between 16 pairs of repeated measures was very high (R = 0.980–1.0), i.e. measurement error was negligible. Body size was significantly decreased in infected colonies in comparison to uninfected ones (ß = − 0.038, SE = 0.014, t = − 2.66, P = 0.013, Fig. 1). We found no significant effect of age (ß = − 0.003, SE = 0.005, t = − 0.65, P = 0.514) or thalli number (ß = 0.0004, SE = 0.006, t = 0.06, P = 0.950, Fig. 2) on body size among infected colonies. Variance inflation factor was 1.025 for both independent variables, suggesting no substantial multicollinearity. Nevertheless, based on the Poisson GLMM, age had a significant positive effect on thalli number (ß = 0.405, SE = 0.011, t = 35.71, P < 0.001, Fig. 3). Furthermore, we found no significant difference between trait correlation matrices of uninfected and infected colonies, indicating that they exhibit the same allometries (Χ^2^ = 1.313, P = 0.999, Fig. 4).

## Discussion

Our results show that: *R. wasmannii* infected *M. scabrinodis* workers are significantly smaller in a natural environment than uninfected ones (1), and this decline in body size in the infected population is consistent across all observed characters, but not affected by thalli number (2). It is important to note that we did not detect shifts in allometries in the characters measured (3). The significant decrease in size took place synchronously. The lack of shifts in static trait allometries indicates that the size decline in the next generation of workers is not ascribed to physiological stress due to direct larval infections but to a colony-level stress effect caused by widespread infection of *R. wasmannii* fungus across the colonies’ adults. Discrete factors, such as parasitism, are known to modify larval development through altered physiological processes, and these developmental perturbations leave their mark on the allometry of some traits of subsequent adults^29^, but no traces of such parasitism are detected in natural *Myrmica* populations. We suppose that a colony-level stress in response to *R. wasmannii* infection is mediated toward larvae by nursing workers, resulting in significantly smaller static trait size among the next generation of their nest mates.

Understanding the colony-level effect of *R. wasmannii* infection on *Myrmica* colonies in a natural environment would foster a better understanding of the dynamics of this ectoparasitic fungus and the host-parasite system. This issue has interesting implications, because *Myrmica* ants are a known host species of a guild of ant guests and socially parasitic organisms which live together with their colonies^8,30^. This network is very sensitive, and every single component of this very complex system might have a regulatory role of its own. Our research is the first undertaking that provides quantitative evidence concerning a decline in worker size in infected nests, and reveal that chronic *R. wasmannii* infection has a long, intergenerational, detrimental colony-level impact in the natural environment.

Infected colonies can somehow manage to compensate for the negative effects of *R. wasmannii* infection (colonies operate with queens, larvae and pupae and they have the capacity to produce sexual forms and maintain their populations), but certain functions are clearly impaired, which is reflected in the decrease in body size among the next generation of workers.

The background of the workers’ mediated colony-level stress as a consequence of parasitism is not entirely clear. Similarly to other members of the order Laboulbeniales, *R. wasmannii* does not penetrate the cuticle of the host, so the most likely hypothesis concerning the feeding of the parasitic fungus is that it absorbs the necessary nutrients from the workers’ cuticle surface or directly from the environment^31^. Ants have numerous exocrine glands, the secretions of which are spread on the cuticle surface by self-grooming and allogrooming. This may be confirmed by the fact that infected *M. scabrinodis* workers show increased sanitary behavior^14^. This behavior has been observed in the invasive garden ant (*Lasius neglectus* van Loon boomsma et Andrásfalvy, 1990) infected by *Laboulbenia formicarum* Thaxt.^32^, where fungus also was not found to penetrate the cuticle of its hosts^31^. This increased sanitary behavior could mean that ant workers have less time and energy to care for and feed the brood, which could be another explanation for the decline in body size in subsequence generations in infected colonies.

We also speculate that the decline in size among workers in infected *Myrmica* ant colonies is part of the colony-level strategy to minimize the adverse effects of the infection by maintaining the number of workers at the cost of smaller size rather than producing fewer, but “normal size” workers. It can be a part of a counter adaptation in which the reduced size results in cheaper workers, offsetting the negative effects of infection. Previous studies have shown that infected individuals have a shorter lifespan (reduction of the outside workforce) and this could lead to reduced food intake, which could result in smaller workers^33^. Production of smaller, but still operational workers may spare resources and “economically optimized worker production” for counter adaptation to infection might also provide an alternative explanation. From an evolutionary biological perspective, whether a *R. wasmannii* infection impacts the fitness of *M. scabrinodis* colonies is an important question. We do not know whether infected colonies produce smaller or perhaps fewer sexuals or this ultimate function is not impaired. This topic merits further investigation.

## Materials and methods

### Study site

**Figure 5:**
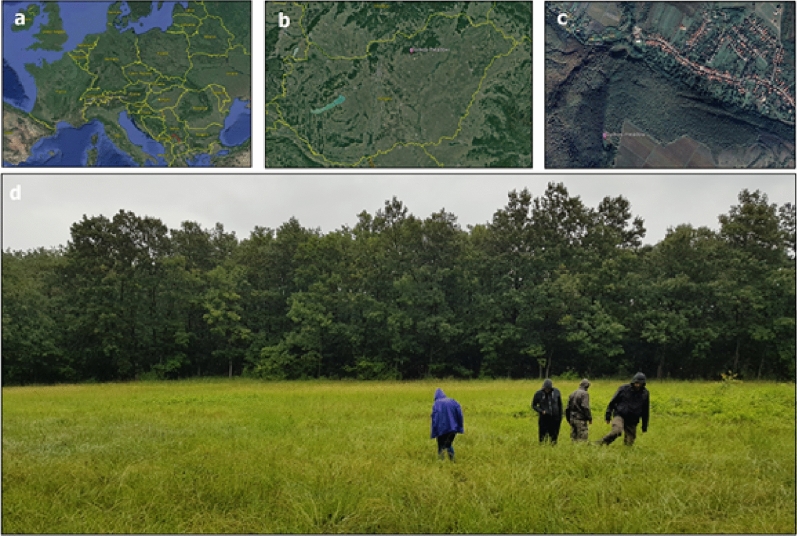
The field site where *M. scabrinodis* workers for our study were collected. Field site is located in Europe (**a**), Hungary (**b**) near Pálosvörösmart (**c**). Wet meadow (Gyilkos-rét) surrounded by deciduous forest (**d**) Figure is created by using Google Earth Pro ver. 7.3.3.7786 (https://www.google.hu/intl/hu/earth/versions/#earth-pro) and Microsoft Paint ver. 2004 (https://support.microsoft.com/hu-hu/windows/a-microsoft-paint-megnyit%C3%A1sa-ead1dc5c-abc4-fd2c-d81e-ebb013fbc113).

Material for the present study was collected from a relatively small, one-acre fragment of a natural marshland surrounded by deciduous, oak-dominated forest near Gyöngyös, Sár-hegy: Gyilkos-rét (23.06.2016; 47.811206, 19.988027; 320 m a.s.l) in Hungary (Fig. 5). This area is part of the Bükk National Park and has not been disturbed or extensively cultivated over the course of the past two decades.

### Sampling

**Figure 6:**
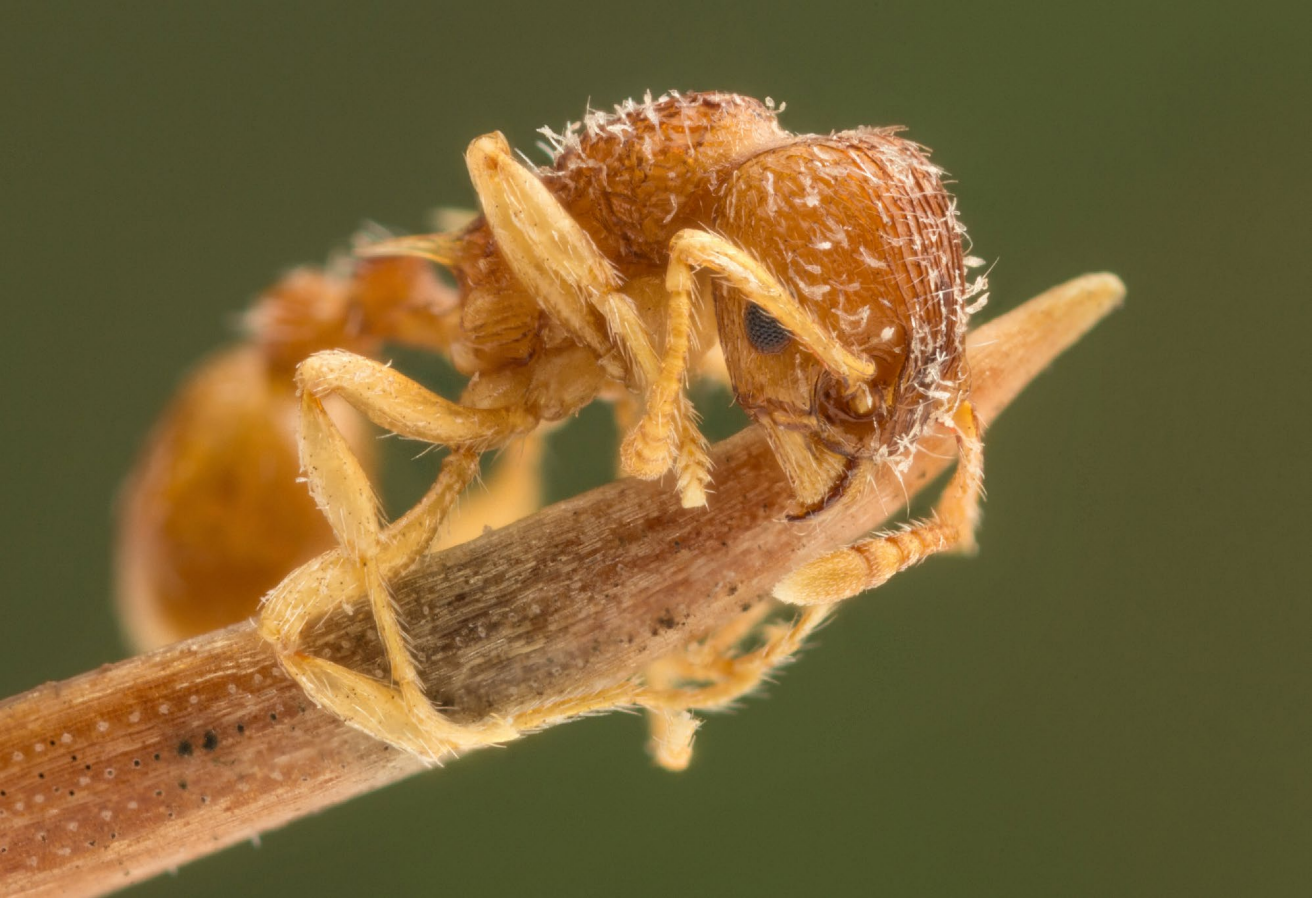
Habitus image of an infected *Myrmica scabrinodis* worker. *Rickia wasmannii* thalli cover the head, mesosoma, and, to a lesser extent, the appendages. Photo by Zsolt Ujvári.

Infected (Fig. 6) and uninfected *M. scabrinodis* colonies were found via hand searching in the grassland by FB and AT. Sampling activities were concentrated in a short, one-day period of time (23.06.2016) because *R. wasmannii* infection is known to show seasonal fluctuation^26^. Altogether 30 *M. scabrinodis* colonies (15 infected and 15 uninfected ones) were sampled, and 15–15 specimens were taken from each colony. The nests were carefully opened and the presence of *R. wasmannii* on the workers’ body surfaces was checked in every colony with the use of a 40× magnifying glass in the field. The sampled workers were stored in 1.5 mL Eppendorf tubes with 67.5% EtOH until observation in the laboratory.

### Thallus number on the workers’ cuticular surfaces

Altogether, 225 *M. scabrinodis* workers (15 individuals from each infected colony) were screened (by FB) for fungal thalli (Fig. 6). All fungal thalli on the whole ant body were counted using a Leica MZ125 stereomicroscope at × 10–160 magnification as described by Báthori et al.^27^. After the fungal thalli had been counted, colony infection level was calculated based on arithmetic mean thalli number of 15 randomly sampled workers from each colony.

### Estimating worker age

As was done by Báthori et al.^27^, all infected *M. scabrinodis* workers screened for fungal thalli were separated into different age groups. Based on the degree of cuticular pigmentation, five different age categories were described by Cammaerts-Tricot^34^. The infected individuals were classified into categories according to cuticle coloration from younger to older (1–5). The highly pigmented oldest workers were given the highest numbers.

### Morphometric character recording

The measured morphometric characters are defined as in Csősz and Majoros^29^. The measurements of altogether 300 *M. scabrinodis* workers (10 from each colony) were made with an ocular micrometer using a Leica MZ125 stereomicroscope at a magnification of × 50 for CL, CW and FR, × 100 for FL, SL and PPW (all measurements were recorded in μm). All measurements were made by FB. Measured characters are defined in Table 1. Raw data are available in Supplementary Table 1.

**Table 1:** Verbatim trait definitions for morphometric character recording.

Abbr.	Description of traits	ICC
CL	Cephalic length measured from the anterior-most point of clypeal margin to the mid-point of the occipital margin, in full-face view	0.990 [0.980, 1.000]
CW	Cephalic width measured in full-face view, including compound eyes	0.997 [0.994, 1.000]
FR	Frons width measured according to the minimum distance between the frontal carinae	0.995 [0.990, 1.000]
FL	Frontal lobe width measured according to the maximum distance between external borders of the frontal lobes	0.995 [0.991, 1.000]
SL	Scape length measured from the neck to the distal end of the scape	0.993 [0.986, 1.000]
PPW	Postpetiole width measured according to the maximum width of the postpetiole in dorsal view	0.996 [0.991, 1.000]

Abbreviation (Abbr.), verbatim character definition and intraclass correlation coefficients (ICC) of certain morphometric traits are provided. Upper and lower bounds of ICC scores, separated by a coma, are also given in parentheses.

### Measurement error assessment

All measurements are subject to error, therefore repeatability, i.e., the degree of agreement between pairs of observations made on the same measurand under the same conditions, i.e. made by the same observer, using the same microscope, following the same measurement protocol as defined in Csősz et al.^35^, was tested before the statistic framework was created. The repeatability of the recorded size parameters was assessed via Intraclass Correlation Coefficients (ICC) on repeated measurements of 16 ant specimens using Package ICC^36^. ICC scores are given for each characteristic in Table 1.

### Statistical analysis

All data analyses were performed with R version 4.0.2^37^. To reduce the number of variables on body size we used principal component analysis (PCA) by non-linear iterative partial least squares (NIPALS) with the R-package “nipals”^38^. We preferred this method over classical PCA because in a small number of cases (7 in total) size measurements of some body parts for a given ant were not feasible (hence the missing measurements); NIPALS can use data with missing observations, whereas in classical PCA we should have excluded all those specimens for which any measurement was missing. Variables were centered at zero (by subtracting variable mean from each value) and rescaled (by dividing all values by the variable standard deviation) in order to bring them to the same scale. We retained only the first PCA axis (being the only axis with an eigen-value higher than 1), which corresponded to 77% of the total variation in the six body measurement variables and was positively correlated with all variables.

To test whether there is a significant difference in body size between infected and uninfected colonies, we used a mixed-effects linear regression model (LMM) with Gaussian error distribution using the R-packages “lme4” and “lmerTest”^39,40^, specifying the abovementioned PCA axis representing body size as the dependent variable and infection as independent factor. To test how the severity of fungal infection and age affected body size, we fitted another LMM (naturally, only using data from infected colonies) with the body size PCA axis as a dependent variable and the log-transformed number of thalli and age as independent variables. Because previously it had been shown that age and thalli number can be correlated, we estimated variance inflation factors to see whether there is substantial multicollinearity between the independent variables using the “car” package^41^. In addition, we tested the association between age and thalli number using a Poisson generalized LMM (GLMM), specifying thalli number as a dependent variable. In both LMMs and in the GLMM, colony number was used as a random effect to control for the non-independence of observations from the same colonies.

Furthermore, to see whether the infection causes changes in static trait allometries across the measured body size indices, we estimated trait correlations (Pearson’s ρ) separately for uninfected and infected colonies and compared the resulting two correlation matrices^42^.

## Supplementary Information

Supplementary Table 1.

## Data Availability

Raw data are available from the Supplementary Table 1.
